# Comparative Transcriptome Analysis Provides Insights into the Resistance in Pueraria [*Pueraria lobata (Willd.) Ohwi*] in Response to Pseudo-Rust Disease

**DOI:** 10.3390/ijms23095223

**Published:** 2022-05-07

**Authors:** Xinlu Huang, Xiaoxi Huang, Lijun Guo, Longfei He, Dong Xiao, Jie Zhan, Aiqin Wang, Renfan Liang

**Affiliations:** 1 National Demonstration Center for Experimental Plant Science Education, College of Agriculture, Guangxi University, Nanning 530004, China; 1917301013@st.gxu.edu.cn (X.H.); 2017301018@st.gxu.edu.cn (X.H.); 2117401014@st.gxu.edu.cn (L.G.); lfhe@gxu.edu.cn (L.H.); xiaodong@gxu.edu.cn (D.X.); may2399@163.com (J.Z.); 2 Key Laboratory for Agro-Environment and Agro-Product Safety, Nanning, Guangxi University, Nanning 530004, China; 3 Key Laboratory of Crop Cultivation and Tillage, Guangxi University, Nanning 530004, China; 4 Academy of Agricultural Science, Guangxi University, Nanning 530004, China

**Keywords:** Pueraria [*Pueraria lobata (Willd.) Ohwi*], *Synchytrium puerariae Miy*, pseudo-rust disease, transcriptome analysis, differentially expressed genes

## Abstract

*Pueraria lobata* is an important medicinal and edible homologous plant that is widely cultivated in Asian countries. However, its production and quality are seriously threatened by its susceptibility to pseudo-rust disease. The underlying molecular mechanisms are poorly known, particularly from a transcriptional perspective. Pseudo-rust disease is a major disease in pueraria, primarily caused by *Synchytrium puerariae Miy* (SpM). In this study, transcriptomic profiles were analyzed and compared between two pueraria varieties: the disease-resistant variety (GUIGE18) and the susceptible variety (GUIGE8). The results suggest that the number of DEGs in GUIGE18 is always more than in GUIGE8 at each of the three time points after SpM infection, indicating that their responses to SpM infection may be different, and that the active response of GUIGE18 to SpM infection may occur earlier than that of GUIGE8. A total of 7044 differentially expressed genes (DEGs) were identified, and 406 co-expressed DEGs were screened out. Transcription factor analysis among the DEGs revealed that the bHLH, WRKY, ERF, and MYB families may play an important role in the interaction between pueraria and pathogens. A GO and KEGG enrichment analysis of these DEGs showed that they were mainly involved in the following pathways: metabolic, defense response, plant hormone signal transduction, MAPK signaling pathway-plant, plant pathogen interaction, flavonoid biosynthesis, phenylpropanoid biosynthesis, and secondary metabolite biosynthesis. The CPK, CESA, PME, and CYP gene families may play important roles in the early stages after SpM infection. The DEGs that encode antioxidase (CAT, XDH, and SOD) were much more up-regulated. Defense enzyme activity, endogenous hormones, and flavonoid content changed significantly in the two varieties at the three infection stages. Finally, we speculated on the regulatory pathways of pueraria pseudo-rust and found that an oxidation-reduction process, flavonoid biosynthesis, and ABA signaling genes may be associated with the response to SpM infection in pueraria. These results expand the understanding of pueraria resistance and physiological regulations by multiple pathways.

## 1. Introduction

Pueraria [*Pueraria lobata (Willd.) Ohwi*], a perennial vine and an important species of Pueraria DC [1], is a kind of medicinal plant peculiar to China, with distinguishing features such as rapid growth, high biological yield, good adaptability, and strong resistance to environmental stresses [2,3]. For hundreds of years, pueraria has been thought to be an important Chinese traditional medicine and homologous food with proper marketing potential. Its roots are not only rich in nutrients [4,5], but they also possess many pharmacological properties including flavonoids and isoflavones [6], which have been widely used for the treatment of and protection from various illnesses [7]. Also known as pueraria rust disease, pseudo-rust is one of the most destructive diseases for pueraria and has resulted in huge economic losses to the pueraria industry. Pueraria pseudo-rust was first discovered by Feng et al. in Guangzhou, China, in 1995. After identification, it was found that pueraria pseudo-rust was a fungal disease caused by *Synchytrium puerariae Miy* [8], which mainly affects leaves, petioles, and kudzu vines.

Although researchers have made efforts to study the pueraria disease, available achievement reports are limited [9,10]. Flavonoids and puerarin, products of the flavonoid biosynthesis pathway, are important indexes to measure the medicinal quality of pueraria, which has been confirmed to improve disease resistance in plants [11,12]. The flavonoid synthesis pathway of pueraria has been gradually explored by researchers [13,14]. However, pseudo-rust disease and the underlying molecular mechanisms are poorly known, particularly from a transcriptional perspective. RNA sequencing (RNA-Seq), as a revolutionary technique for transcriptome analysis, has been widely used to identify core pathways and responsive genes under biotic or abiotic stress. It provides a precise measurement for transcript levels to reveal the response mechanisms for specific stimuli.

The large-leaf medicinal type, GUIGE18, is a highly resistant cultivar, with a disease index (ID) of 0–0.16 and an average ID of 0.04. The large-leaf processing type, GUIGE8, is a moderately strong, but susceptible, cultivar, with an ID of 18.00–50.31 and an average ID of 31.98. It has been proven that the disease severity of GUIGE18 is significantly lower than that of GUIGE8 [15]. Meanwhile, there are significant differences in phenotypic characteristics and quality indicators between the two cultivars [16]. In this study, a comparative transcriptome analysis of the resistant cultivar (GUIGE18) and the susceptible cultivar (GUIGE8) was performed to identify some candidate DEGs that may be related to pseudo-rust resistance. The results may provide several new insights into the molecular defense mechanisms of pueraria cultivars exhibiting a strong resistance to SpM infections, along with valuable information for breeding resistant pueraria cultivars.

## 2. Results

### 2.1. Transcriptome Analysis

To obtain a comprehensive understanding of gene expression dynamics in leaves of pueraria after SpM infection, cDNA libraries from a total of 18 samples of resistant and susceptible cultivars were subjected to transcriptome sequencing at 0, 1, and 3 days, post SpM infection. The resistant cultivar (GUIGE18) is denoted by GL_18CK, GL_183D, and GL_181D, respectively, where G stands for GUIGE, L stands for leave, CK stands for control, and the number indicates the sampling time post SpM infection. The susceptible cultivar (GUIGE8) is denoted by GL_8CK, GL_81D, and GL_83D, respectively. The same naming method as above was used.

The RNA-Seq analysis of 18 cDNA libraries generated an average of 6.5 Gb of high-quality reads, characterized by Q20-values (≥98.00%) and a Q30-values (≥94.90%) (Supplementary Table S1). Since the full pueraria genome has not been published, a strategy of unreferenced transcriptome analysis was used, namely the de novo assembly of sequenced reads using Trinity. A total of 183,472 transcript and 70,896 unigenes were obtained. These had an N50 value of 1678 bp and an N50 value of 1493 bp, respectively (Supplementary Figure S2). A correlation analysis of the expressing materials for all samples showed that the three biological replicates from each treatment were correlated (Supplementary Figure S3). To verify whether the gene expression in the transcriptome was accurate, nine unigenes participating in different pathways were chosen randomly for a qRT-PCR analysis. The relevant PCR primer sequences are shown below (Supplementary Table S2), and were designed based on the CDS sequences in the transcriptome sequencing by the Primer 5. The expression levels of the nine unigenes were all consistent between the qRT-PCR analysis and RNA-Seq data, which demonstrated that the RNA-seq sequencing results have high accuracy (Supplementary Figure S4). The sequencing data and quality meet the requirements of further analysis.

### 2.2. Overview of Functional Annotation

**Figure 1:**
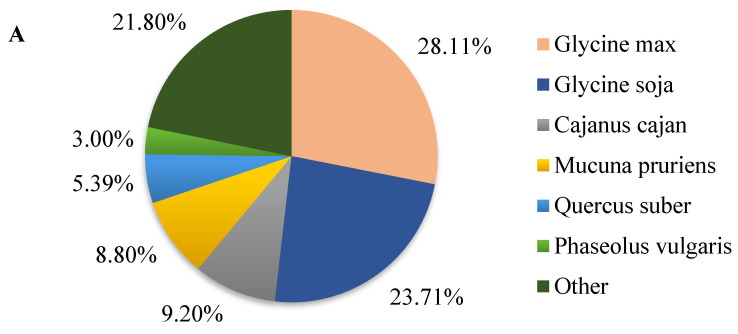

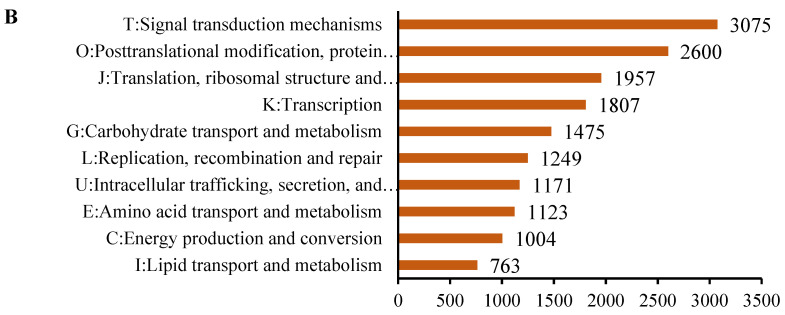
NR functional and eggNOG classified annotation of transcriptome genes. (**A**) Species homology distribution analysis of unigenes in NR database. The ratio indicates the number of unigenes that match to the homologous species/a total of unigenes—the larger the value, the higher the homology; (**B**) the eggNOG classified annotation analysis of genes (only the top 10 enriched items are shown). The text in the left column represents the name of the enriched item in the eggNOG database, and the values indicate the number of genes in each enriched item.

Of the 70,896 unigenes, 45.57%, 35.97%, 42.35%, 36.86%, 55.77%, 35.97%, and 56.18% were matched to the GO, KEGG, Pfam, Swissprot, eggNOG, KOG, and NR databases, respectively. A total of 59,701 (84.21%) unigenes were mapped to at least one public database, while 10.50% unigenes (7445) were co-annotated in all seven databases (Table 1). A species distribution analysis in the NR database showed that unigenes had the highest homology to *Glycine max* sequences, accounting for 28.11% (11,196 unigenes), followed by *Glycine soja* (23.71%; 9444 unigenes), *Cajanus cajan* (9.20%; 3663 unigenes), *Mucuna pruriens* (8.80%; 3504 unigenes), *Quercus suber* (5.39%; 2147 unigenes), and *Phaseolus vulgaris* (3%; 1193 unigenes). The remaining 21% of the unigenes was annotated to other species, which accounted for less than 3% of each species (Figure 1A). The functional annotation analysis of 39,537 known genes detected, showed that only 20,053 genes were effectively annotated in the eggNOG database. The top ten homologous clustering annotated genes were listed. Topping the list was the signal transduction mechanism, accounting for 15.33% (Figure 1B). These results indicate that the signal transduction mechanism may play an important role in the resistance mechanism of pueraria in response to pseudo-rust.

### 2.3. RNA-Seq Analysis of the Two Cultivars at the Early Stage after SpM Infection and Identification of Differentially Expressed Genes (DEGs)

**Figure 2:**
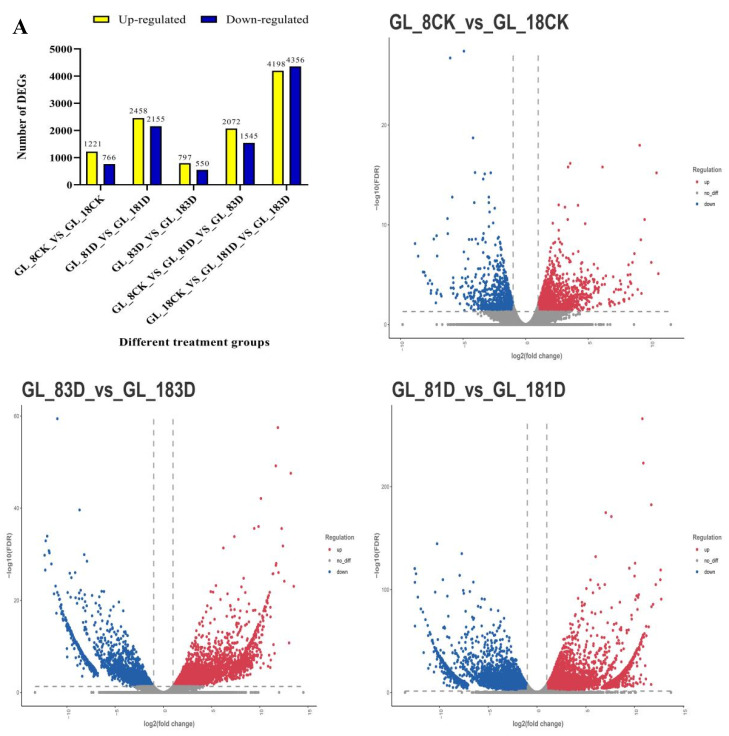

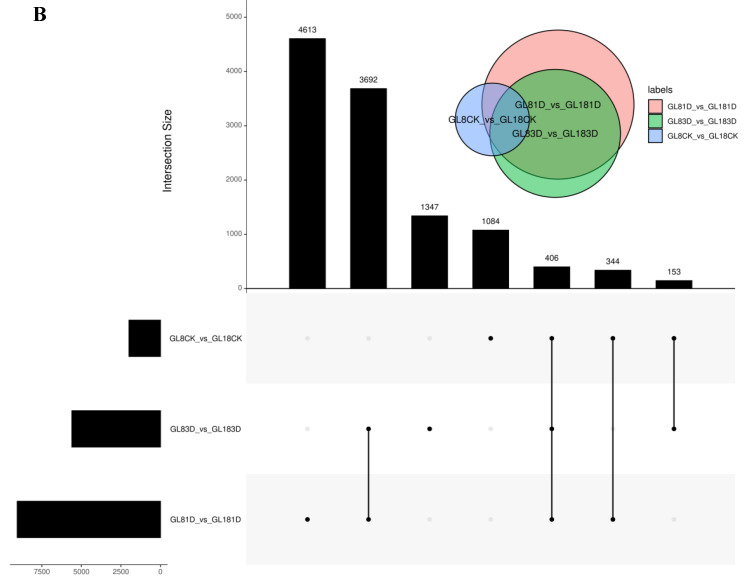
Differentially expressed genes (DEGs) across three time points after SpM infection in resistant (GUIGE18) and susceptible cultivars (GUIGE8). (**A**) Distributions of up-regulated and down-regulated DEGs between two cultivars across the three time points after SpM infection. The colors are shaded according to the log10(FDR)-values level in volcano plots. Red stands for up-regulated genes, blue stands for down-regulated genes, and grey stands for no-difference genes. The number of the circle indicates the number of genes. (**B**) the statistics of DEGs between two cultivars across the three time points after SpM infection.

To further identify the genes closely correlated with the phenotypes of the two varieties, differentially expressed genes (DEGs) were further obtained by comparing their expression levels in GUIGE18 and GUIGE8 at three infection stages. Compared to control plants with sterile water (CK), DEGs were identified in both cultivars at different time points post SpM inoculation (Figure 2). A total of 3617 DEGs were found in the susceptible cultivar (GUIGE8) and total of 8554 DEGs were found in the resistant cultivar (GUIGE18) in the three treatment groups **(**Figure 2A). In GUIGE8, 2072 DEGs were up-regulated and 1545 DEGs were down-regulated. In GUIGE18, 4198 DEGs were up-regulated and 4356 DEGs were down-regulated. During the sampling period of 0–3 days, the number of DEGs in GUIGE18 was higher than in GUIGE8. While there were more down-regulated genes than up-regulated in GUIGE18, GUIGE8 was the opposite (Figure 2A).

The DEGs were also analyzed between the two cultivars at the same time points after SpM inoculation. Amounts of 1084, 4613, and 1347 DEGs were specifically expressed in the GL_8CK vs GL_18CK, GL_81D vs GL_181D, and GL_83D vs GL_183D comparisons, respectively (Figure 2B). Furthermore, 1221, 2458, and 797 genes were up-regulated, while 766, 2155, and 550 genes were down-regulated in the comparable group, respectively. There were more up-regulated genes than down-regulated in all comparisons (Figure 2A). Finally, a total of 406 DEGs were expressed across all three comparisons (Figure 2B). The number of DEGs increased significantly on the first day after SpM inoculation, which was the largest of all treatments in the two cultivars. This showed that the genes could respond quickly with resistance to pueraria pseudo-rust in the early stage after SpM infection.

### 2.4. GO Function Enrichment Analysis of DEGs

To further identify the function of the notable transcripts that are differentially expressed between the two cultivars under SpM infection, we performed a GO enrichment analysis of the DEGs from the three comparisons.

**Figure 3:**
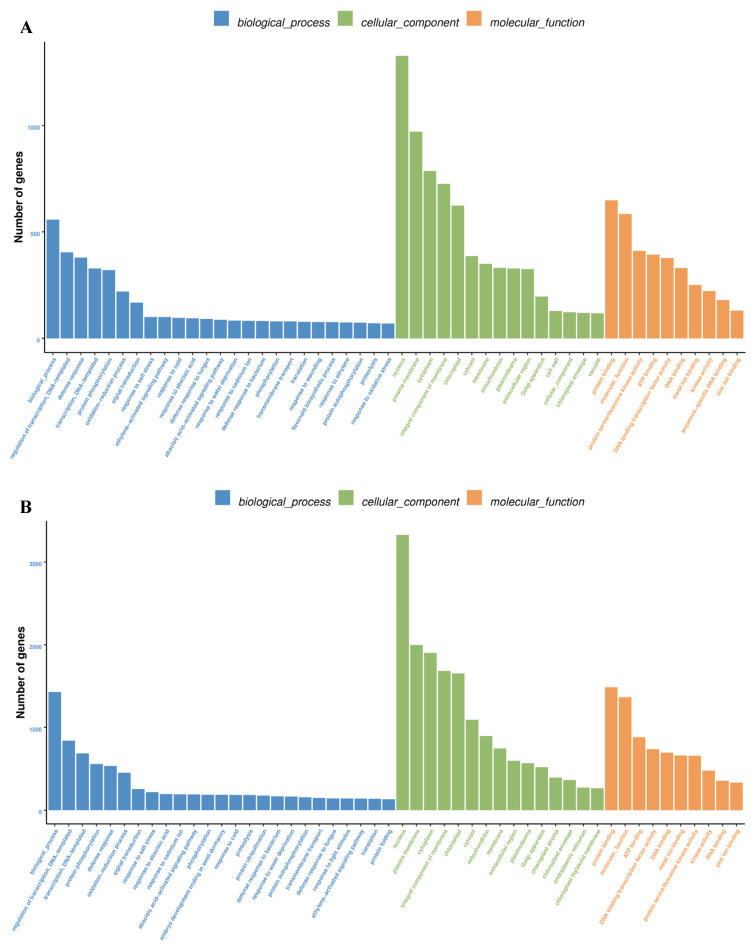

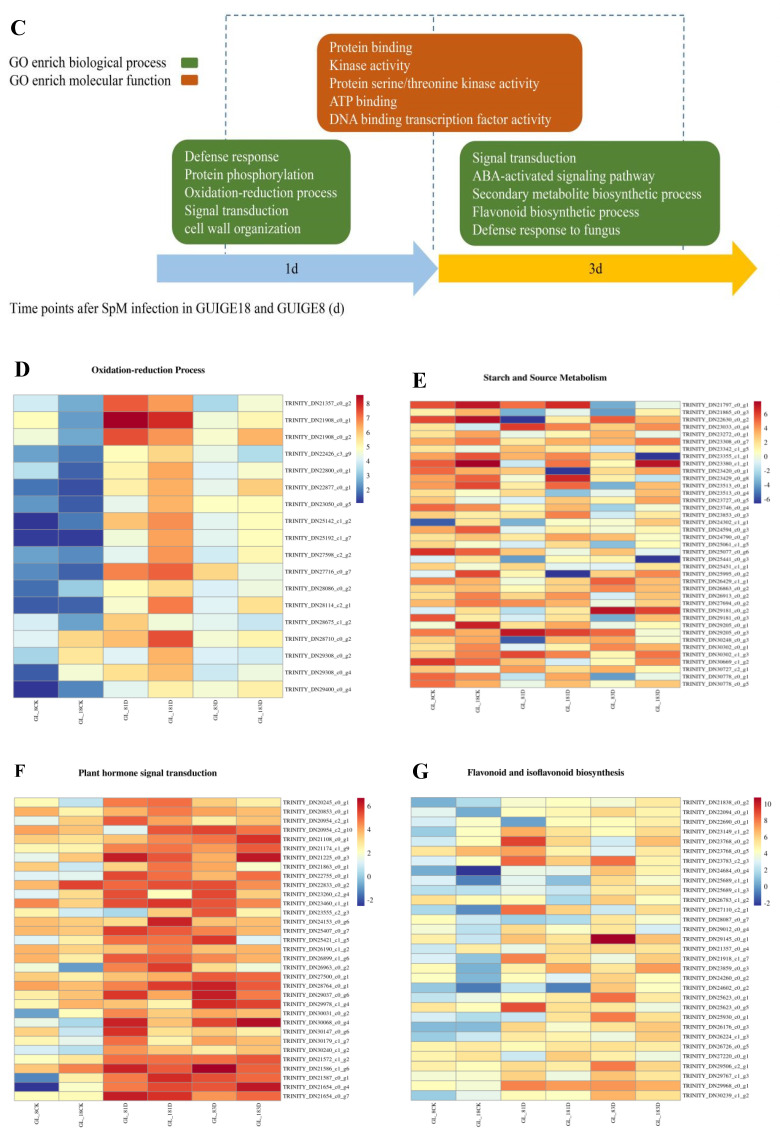
Gene Ontology (GO) enrichment and expression heatmap analysis of DEGs. (**A**) GO enrichment analysis of DEGs in GUIGE8; (**B**) GO enrichment analysis of DEGs in GUIGE18−only the top 25 biological process (BP), 15 cellular component (CC), and 10 molecular function (MF) terms in each cluster are shown; (**C**) GO categories of up−regulated DEGs were significantly enriched in the BP and MF terms at different time points after SpM inoculation in the two cultivars; (**D**) expression heatmap analysis of DEGs related to the oxidation-reduction process; (**E**) expression heatmap analysis of DEGs related to starch and source metabolism; (**F**) expression heatmap analysis of DEGs related to plant−hormone signal transduction; and (**G**) expression heatmap analysis of DEGs related to flavonoid and isoflavonoid biosynthesis. The colors are shaded according to the log10−values level, as shown in the color bars changing gradually from low (blue) to high (red).

In GUIGE8, the most abundant GO biological process terms were the regulation of transcription, DNA-templating (GO: 0006355), defense responses (GO: 0006952), protein phosphorylation (GO: 0006468), oxidation-reduction processes (GO: 0055114), signal transduction (GO: 0007165), abscisic-activated signaling pathways (GO: 0009738), and flavonoid biosynthetic processes (GO: 0051555), in which gene expression increased from day 0 to 1, and high expression levels were maintained thereafter (Figure 3A). Molecular function analysis showed that these target genes were mainly enriched in protein binding (GO: 0005515) and kinase activity (GO: 0016301). In GUIGE18, the DEGs involved in transcription, DNA-templating (GO: 0006351), protein phosphorylation (GO: 0006468), defense responses (GO: 0006952), signal transduction (GO: 0007165), responding to abscisic acid (GO: 0009737), translation (GO: 0006412), and responding to bacterium (GO: 0009617) were enriched in GO biological process terms (Figure 3B). According to GO analysis, although the enrichment items of the two varieties were similar, the enrichment of time and number were different. From day 0 to 1, the number of DEGs in the three functional items increased significantly, while it had a visual reduction from day 1 to 3. The results showed that more biological process related DEGs were significantly enriched earlier in GUIGE18, indicating that GUIGE18 responded faster than GUIGE8. The DEGs expression levels of 4 representative enriched function items and pathways were analyzed and the heatmaps were illustrated. We found that DEGs related to oxidation-reduction processes, along with those related to starch and source metabolism, have higher expression levels in prophase after SpM infection (Figure 3D,E), while DEGs related to plant-hormone signal transduction, and flavonoid and isoflavonoid biosynthesis, were expressed more actively in anaphase (Figure 3F,G).

The GO terms with up-regulated gene enrichment in the two cultivars were summarized (Figure 3C), the GO enrichment results for DEGs between GUIGE8 and GUIGE18 at each time point also remained mostly consistent with the above results, indicating that these DEGs were actively expressed after SpM infection.

### 2.5. KEGG Pathway Analysis of DEGs

**Figure 4:**
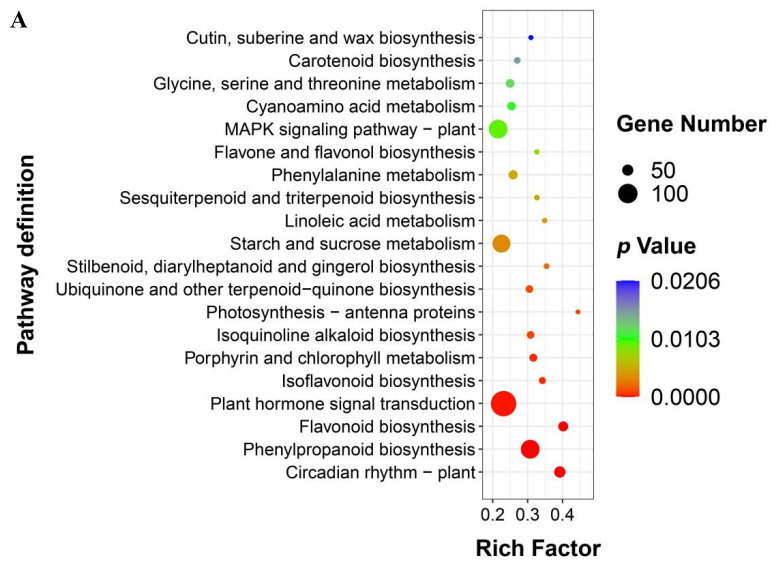

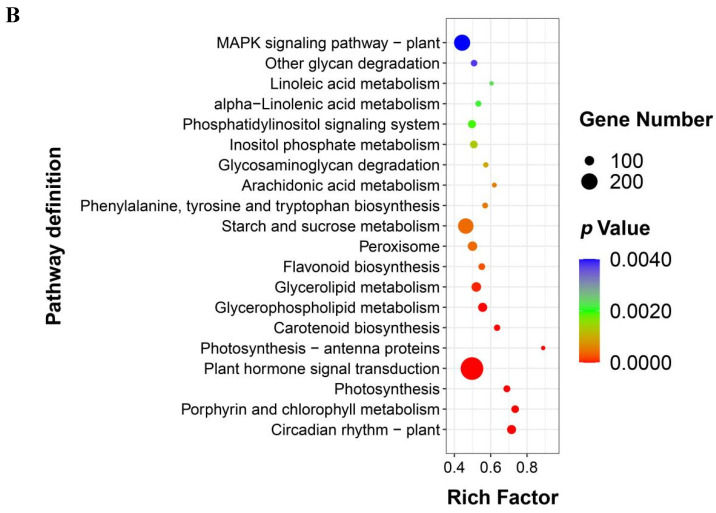
Kyoto Encyclopedia of Genes and Genomes (KEGG) pathway enrichment analysis of DEGs. (**A**) KEGG pathway enrichment analysis of DEGs in GUIGE8; (**B**) KEGG pathway enrichment analysis of DEGs in GUIGE18. The colors are shaded according to the *p*-value level, as shown in the color bars gradually changing from low (red) to high (blue). The size of the circle indicates the number of DEGs from small (less) to big (more). The larger the rich factor, the higher the GO enrichment degree.

To further elucidate the functions of the DEGs in response to pseudo-rust, the Kyoto Encyclopedia of Genes and Genomes (KEGG) databases were used to classify DEGs into corresponding pathways. Many KEGG pathways were significantly enriched in the two cultivars. The KEGG enrichment analysis results are shown as a scatter plot (Figure 4). In our study, plant hormone signal transduction (map04075), MAPK signaling pathway-plant (map04016), starch and source metabolism (map00500), and flavonoid biosynthesis (map00941) were the high enriched pathway in both cultivars, containing 292, 197, 187, and 59 DEGs in GUIGE18, and 136, 96, 91, and 58 DEGs in GUIGE8, respectively. The results show that although there were similarities in the participating pathways, the expression changes of most DEGs involved in the pathways showed different dynamic trends in both cultivars, and the increased expression of GUIGE18 was significantly stronger than that of GUIGE8 on 1D and 3D after SpM infection. The differences in the enrichment and expression trends of the DEGs between the two cultivars can be important reference factors when selecting resistant gene candidates. Moreover, if one gene is expressed significantly in GUIGE18, but not in GUIGE8 in the same pathway, that gene was reasonably speculated to be the valuable research target responding to pseudo-rust resistance in GUIGE18.

### 2.6. DEGs Related to Oxidation-Reduction Process

After pathogen infection, plants usually increase the activity of defense enzymes to limit pathogen invasion. Therefore, the oxidation-reduction process (GO: 0055114) associated DEGs were analyzed. According to the GO enrichment analysis, protein eceriferum 3 (CER3), dioxygenase extradiol-like protein (LIGB), glutathione-S-transferase (GST3), cationic amino acid transporter 1 (CAT1), xanthine dehydrogenase 2 (XDH2), superoxide dismutase 1 (SOD1), oxidative phosphorylation pathway including cytochrome c oxidase-2 (COX-2), and cytochrome oxidase (COI) were obtained in both cultivars.

**Figure 5:**
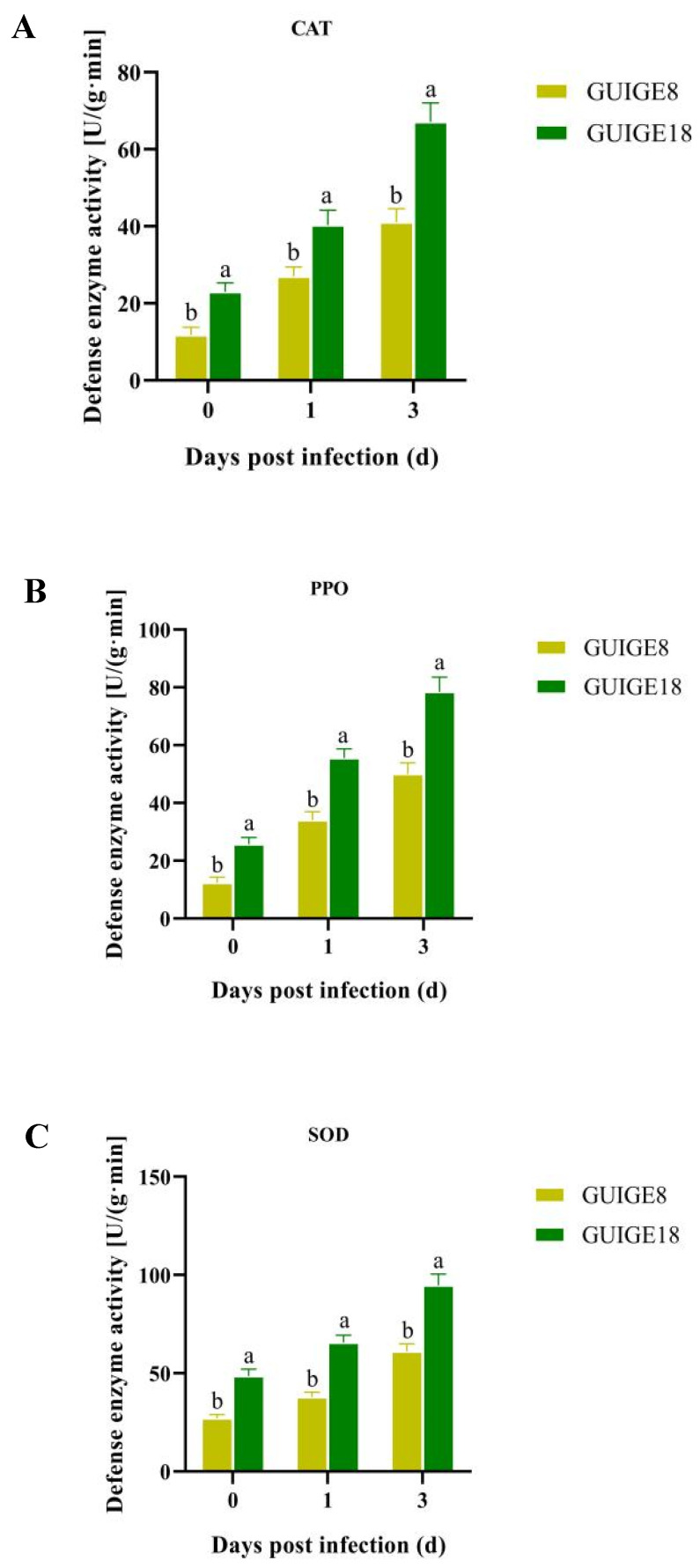
The activity of defense enzymes across three time points (0, 1 and 3 days) after SpM infection in resistant (GUIGE18) and susceptible cultivars (GUIGE8). (**A**) Catalase (CAT); (**B**) Polyphenol oxidase (PPO); and (**C**) Superoxide dismutase (SOD). Letters ‘a/b’ indicate difference changes between the two cultivars on the same day (*p* < 0.05), according to Duncan’s multiple range test using SPSS software.

**Figure 6:**
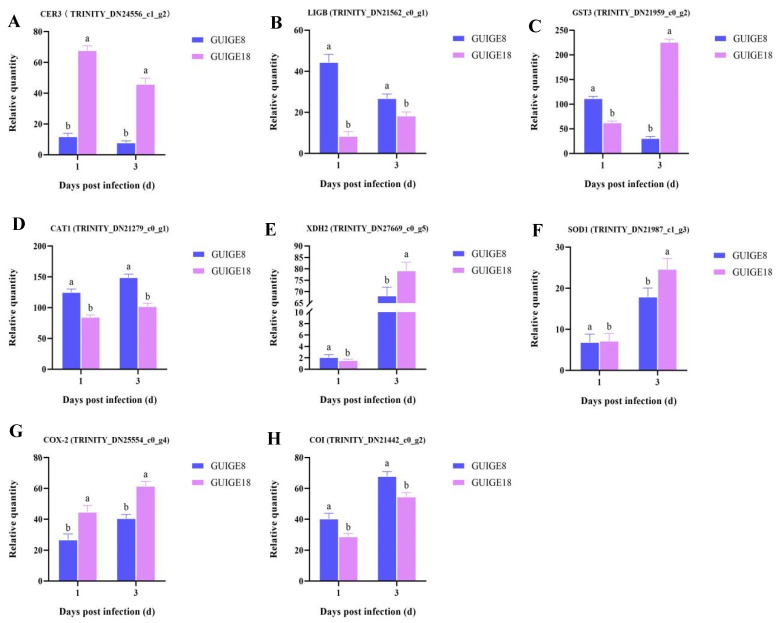
The expression of DEGs related to the oxidation-reduction process. (**A**) CER3; (**B**) LIGB; (**C**) GST3; (**D**) CAT1; (**E**) XDH2; (**F**) SOD1; (**G**) COX-2; and (**H**) COI. The Y axis shows the relative quantity means of three independent replicates, calculated using the 2^−^^△△Ct^ method. Letters ‘a/b’ to indicate difference changes between the two cultivars on the same day (*p* < 0.05), according to Duncan’s multiple range test using SPSS software.

The results show that the activities of four defense enzymes of the two cultivars changed significantly after SpM infection. The superoxide dismutase (SOD), polyphenol oxidase (PPO), and catalase (CAT) activities of the resistant cultivar (GUIGE18) were significantly higher than those of the susceptible cultivar (GUIGE8) in both the control and treatment group, with a bigger increase rate and a longer duration at a higher activity level (Figure 5). Compared with the sensitive cultivar, the resistant one had a faster response speed and higher resistance activity to the SpM infection. To explore the relationship between genes and enzyme activity, the expression of the eight genes mentioned above was analyzed. The results showed that CAT1, XDH2, SOD1, COX-2, and COI were up-regulated (Figure 6D–H), while CER was down-regulated in the two cultivars (Figure 6A). The expression level of GUIGE18 was higher than that of GUIGE8 at 1 and 3 days post infection (dpi). LIGB and GST were down-regulated in GUIGE8, but were up-regulated in GUIGE18 (Figure 6B,C). For example, GST is a key enzyme in the glutathione metabolic process (GO: 0006749). Its expression was remarkably more up-regulated at 3 dpi in GUIGE18 compared to GUIGE8 (Figure 6C), which may result in a greater increase in defense enzyme activity in GUIGE18. In summary, the genes that encode enzymes for the oxidation-reduction process showed similar trends in the two cultivars.

### 2.7. DEGs Related to Starch and Source Metabolism

In the early infection time (0–1 days), there was a significant increase in the expression of β-furan glucosidase invertase A (INVA), sucrose synthase gene (Susy), and hexokinase (HKL3) in the starch synthesis pathway. The cell wall metabolic pathway encoding the cellulose synthase A (CSLA), cellulose synthase B (CSLB) family gene, cellulose synthase D (CSLD3) family gene, cellulose synthase A (CESA), and pectin methylesterase repressor (PMEI13) were significantly up-regulated. From 1 to 3 days post infection, the INVA, HKL3, Susy, and glgC, along with genes related to cell wall metabolism, were down-regulated. Meanwhile, cell wall degradation-related xydlucan endoglycan glycosylase (XTR), pectin methylesterase (PME), binding protein 3 (PDCB3), and callose synthase 3 (CalS3) were activated. The expression of both starch synthesis and cell wall metabolic-related genes showed higher expression levels. Thus, the early up-regulation of the genes involved in starch and source metabolism in GUIGE18 and GUIGE8 may provide useful protection against pueraria pseudo-rust.

### 2.8. Endogenous Hormone Content and Related Gene Expression

**Figure 7:**
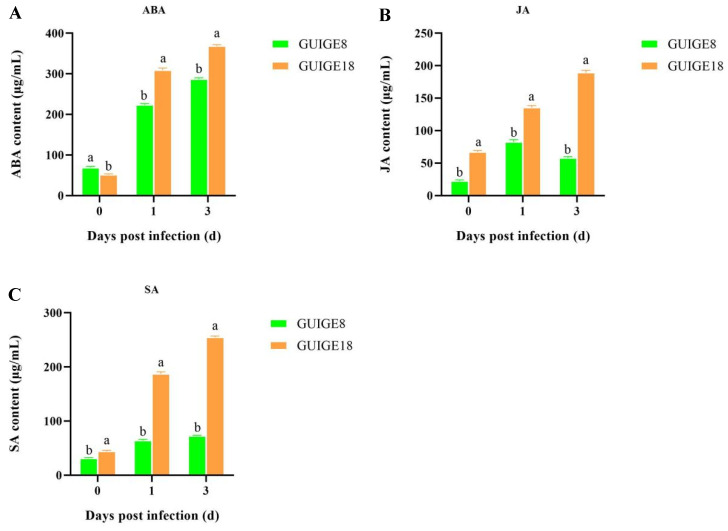
The endogenous hormone content across three time points (0, 1, and 3 days) after SpM infection in resistant (GUIGE18) and susceptible cultivars (GUIGE8). (**A**) Abscisic acid (ABA); (**B**) jasmonic acid (JA); (**C**) salicylic acid (SA). Letters ‘a/b’ indicate difference changes between the two cultivars on the same day (*p* < 0.05), according to Duncan’s multiple range test using SPSS software.

Plant hormones are important for responding to biological stress. As such, the contents of typical endogenous hormones at three infection stages were measured via high-performance liquid chromatography-mass spectrometry (HPLC-MS). The results showed that hormone content increased significantly across the three time points (0, 1, and 3 days) after SpM infection in resistant (GUIGE18) and susceptible cultivars (GUIGE8). As shown in Figure 7, higher JA and SA content was detected in GUIGE18 at all time points, with a maximum level of 189.26 μg/mL and 254.97 μg/mL at 3 days, respectively, which were just over three times higher than those of GUIGE8 (Figure 7B,C). There was also more accumulation of ABA in GUIGE18, which was about two times higher than that of GUIGE8 (Figure 7A).

**Figure 8:**
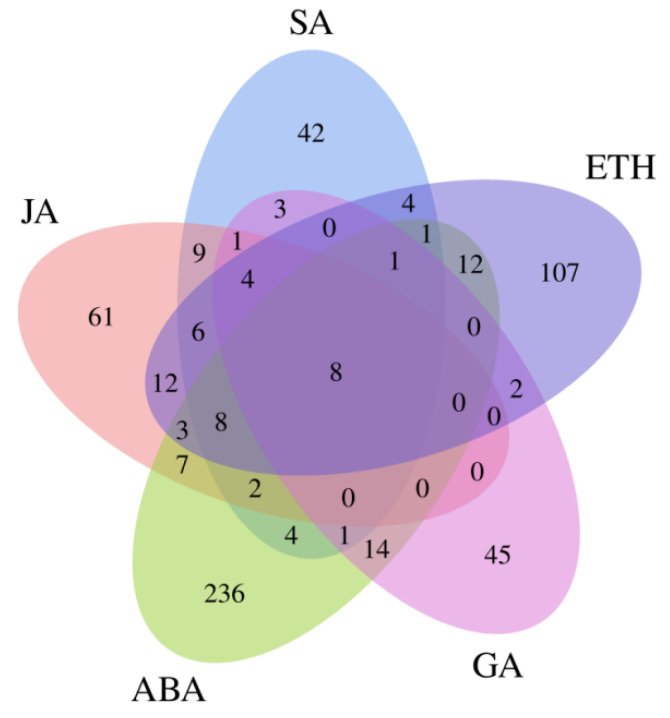
Venn diagram of DEGs in hormone signal transduction pathway across three time points (0, 1, and 3 days) after SpM infection in resistant (GUIGE18) and susceptible cultivars (GUIGE8). JA—jasmonic acid; SA—salicylic acid; ETH—ethylene; GA—gibberellic acid; ABA—abscisic acid.

We found that the largest number of DEGs were involved in the hormone signal transduction pathway. A total of eight co-expressed genes were detected between the ‘GL_81D vs GL_181D’ and ‘GL_83D vs GL_183D’ comparable groups (Figure 8), namely transcription factor MYB44, calcium-dependent protein kinase (CPK20), protein reveille (RVE8), NDR1/HIN1-Like protein (NHL3), protein phosphatase 2C (PP2CA), ABA-responsive element binding factors (ABF4), transcription factor WRKY53, and ethylene-responsive transcription factor (ERF4).

**Figure 9:**
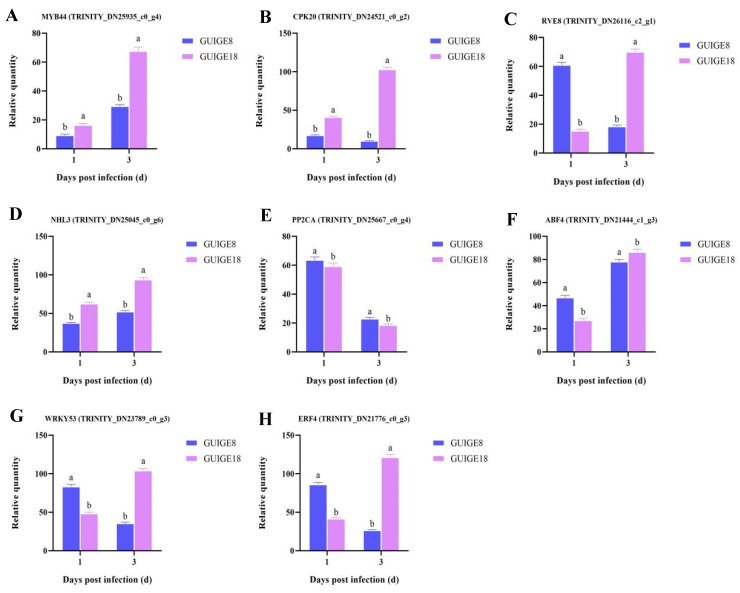
The expression of DEGs related to plant hormone signal transduction. (**A**) MYB44; (**B**) CPK20; (**C**) RVE8; (**D**) NHL3; (**E**) PP2CA; (**F**) ABF4; (**G**) WRKY53; and (**H**) ERF4. The Y axis shows the relative quantity means of three independent replicates, calculated using the 2^−^^△△Ct^ method. Letters ‘a/b’ indicate difference changes between the two cultivars on the same day (*p* < 0.05), according to Duncan’s multiple range test using SPSS software.

The expression of the eight genes was analyzed, and their changes were consistent with the accumulation of hormones (Figure 9). The results showed that MYB44, NHL3, and ABF4 were up-regulated, while PP2CA was down-regulated in the two cultivars. CPK20, RVE8, WRKY53, and ERF4 were down-regulated in GUIGE8, whereas it was up-regulated in GUIGE18. Moreover, CPK20, involved in abscisic-activated signaling pathway (GO: 0009738), was up-regulated in GUIGE18 and was significantly higher than in GUIGE8 at 1 dpi. However, its expression decreased at 3 dpi. ABF4 is a regulatory gene for ABA biosynthesis. Its expression first decreased, and then increased by 3 dpi in both varieties, although the increase was faster in GUIGE18 than in GUIGE8. In summary, the expression dynamics for these genes was consistent with the hormone accumulative dynamics in the two varieties.

### 2.9. Flavonoid Content and Related Gene Expression in Flavonoid Biosynthetic Process

**Figure 10:**
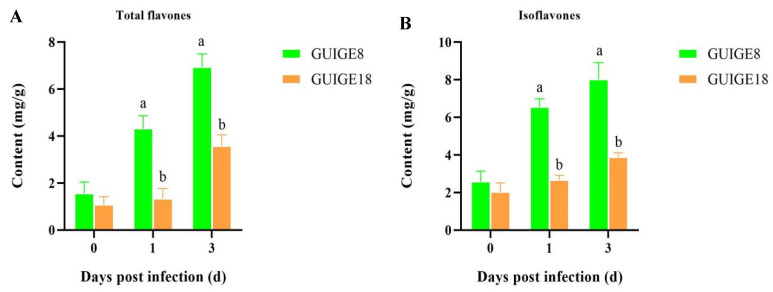
The flavonoid content across three time points (0, 1, and 3 days) after SpM infection in resistant (GUIGE18) and susceptible cultivars (GUIGE8). (**A**) Total flavone content; (**B**) isoflavone content. Letter ‘a/b’ indicate differences change between the two cultivars on the same day (*p* < 0.05), according to Duncan’s multiple range test using SPSS software.

Flavonoids are the main secondary metabolites and phytotoxin substances of pueraria, which significantly change when pueraria is affected by SpM. As the results show, higher total flavone and isoflavone contents were detected in GUIGE8 at all time points with a maximum level of 6.94 and 8.03 mg/g at 3 dpi, which was about twice as high as the levels detected in GUIGE18. The difference is significant, although the increase was faster in GUIGE8 than in GUIGE18 (Figure 10). The total flavone and isoflavone contents in the two varieties showed no differences at 0 dpi, while the contents increased significantly from 1 dpi to 3 dpi (Figure 10A,B). There was also more accumulation in GUIGE8 compared to GUIGE18.

**Figure 11:**
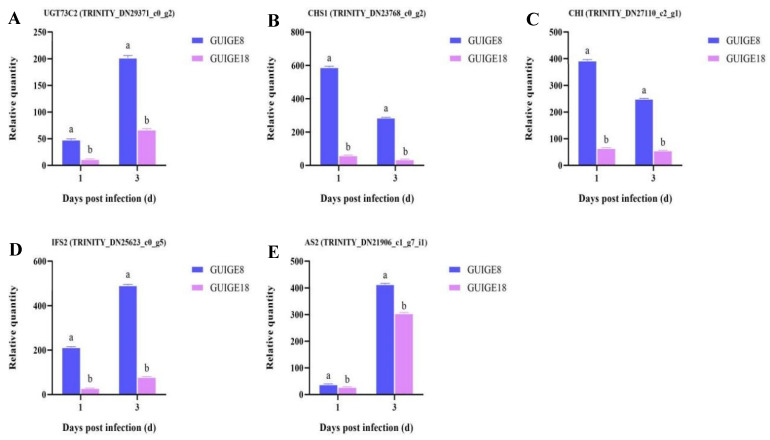
The expression of DEGs related to flavonoid biosynthetic process. (**A**) UGT73C2; (**B**) CHS1; (**C**) CHI; (**D**) IFS2; and (**E**) AS2. The Y axis shows the relative quantity means of three independent replicates, calculated using the 2^−^^△△Ct^ method. Letters ‘a/b’ indicate differences change between the two cultivars on the same day (*p* < 0.05), according to Duncan’s multiple range test using SPSS software.

Secondary metabolite biosynthetic processes (GO: 0044550) play a characteristic and important role in pueraria resistance to disease. Therefore, the expression of the related genes was analyzed. The major genes are shown in Figure 11. The flavonoid biosynthetic process (GO: 0009813), isoflavonoid metabolic process (GO: 0046287), and isoflavonoid biosynthetic process (GO: 0009717) were found to be significantly enriched in GUIGE8. The results showed that UDP-glycosyltransferase (UGT73C2), isoflavone synthase (IFS2), and hydroquinone glucosyltransferase (AS2) were up-regulated, while chalcone synthase (CHS1) and chalcone flavonone isomerase (CHI) were down-regulated in the two cultivars. In addition, CHI, CHS1, and IFS2, which are involved in flavonoid biosynthetic processes, were expressed at a higher level at 1 and 3 dpi in GUIGE8, compared with GUIGE18, in which the levels remained unchanged (Figure 11B,C). For example, IFS2 is a key enzyme in the isoflavonoid metabolic pathway, yet its expression was remarkably more up-regulated at 3 dpi in GUIGE8, compared to GUIGE18 (Figure 11D). The expression of AS2 was low in the two cultivars at 1 dpi, but became ten times more up-regulated at 3 dpi (Figure 11E). In summary, the genes involved in secondary metabolite biosynthetic processes showed similar trends in the two cultivars. Thus, the early up-regulation of the genes in GUIGE8 may provide useful protection against pueraria pseudo-rust.

## 3. Discussion

Studies have shown that plants gradually form a series of defense mechanisms in the long-term interaction and co-evolution with pathogenic microorganisms, which can only be quickly and fully expressed after induction. From the perspective of tissue structure [17,18], physiology, and molecular mechanisms, stress-related genes have different expression patterns at different stages after pathogen infection [19,20,21,22]. Plants respond to stress by altering gene expression levels [23,24]. This phenomenon may be that pueraria regulates physiological metabolism and gene expression in vivo to form the corresponding defense mechanisms. The interrelation between them may be the underlying cause of its disease resistance.

In this study, comparative transcriptome and trend analysis revealed changes in gene expression patterns between resistant and susceptible pueraria cultivars at three different time points after inoculation with the fungal pathogen SpM. Furthermore, some important pathways related to GO and KEGG enrichment were also analyzed.

Cell wall-mediated resistance can be induced through changes in the chemical composition or physical structure of the cell wall after pathogen infection, and is considered to be the first important structural barrier against most pathogens in plants [25,26,27]. Phloem damage and sieve blockage were caused after SpM infection, which may limit the absorption and transport of mineral nutrients and various organic compounds [28]. Cellulase, expandin, and pectin esterase have all been associated with cell wall disruption. In our study, CSLA, CSLB, CSLD3, CESA, and PME, which are involved in the cell wall metabolic pathway, were up-regulated significantly in GUIGE8. The high expression of these genes may be one of the symptoms of sensitive host expression [29,30,31]. In this study, the number of related down-regulated genes increased with the passage of infection time, indicating that the cell wall limiting ability decreased after SpM infection. It was found that INVA, Susy, and HKL3 (involved in starch synthesis) were up-regulated in response to SpM infection.

Reactive oxygen species (ROS) are a system of excited molecules with a certain reactivity, and its eruption plays an important role in disease resistance of plants. In our study, SOD, PPO, and CAT activities increased significantly in both cultivars (Figure 5). Compared with the susceptible cultivar, the resistant one had a faster response speed and higher resistance activity to SpM infection. The oxidation-reduction process was enriched between the two pueraria cultivars. GST3, XDH2, SOD1, COX-2, and COI encoding defensive enzyme had higher expression levels in GUIGE18 at 3 dpi. LIGB and GST were down-regulated in GUIGE8, but were up-regulated in GUIGE18 (Figure 6). The genes encoding enzymes for the oxidation-reduction process showed similar trends in the two cultivars. ROS accumulation may lead to a change in the biofilm fluidity and the inactivation of biological enzymes [32]. A defense system can be formed together with SOD and CAT, which can effectively remove free radicals and peroxides in plants. PPO is related to the oxidation of polyphenols, which can directly affect the polymerization of polyphenol oxides in plants. It is speculated that the increase of defense enzyme activity would cause ROS eruption and change of cell wall composition, which can limit the SpM infection to a certain extent.

Hormone signals usually change significantly when plants are under biotic stress [33]. Systemic acquired resistance (SAR) is one of several induced defense responses in plants [34]. It is regulated by plant hormones responsible for signal transduction and plays a vital role in disease resistance. SA, JA, ABA, ETH, and BRs play important roles in plant–pathogen interaction, and crosstalk between signaling pathways is essential for controlling the immune response during pathogen infection.

In our study, hormone content (ABA, JA, and SA) increased significantly after SpM infection in resistant (GUIGE18) and susceptible cultivars (GUIGE8). However, JA content had a significant decrease at 3 dpi in GUIGE8. Higher content was detected in GUIGE18 at all time points compared to GUIGE8 (Figure 7). The results showed that MYB44, NHL3, and ABF4, which are involved in the hormone signal transduction pathway (map04075), were up-regulated (Figure 9). We found that DEGs that encoded the ABA receptor protein, such as gene PP2CA, was down-regulated after SpM infection in the two cultivars (Figure 9B,E). PP2C family members are key components of the ABA signal transduction pathway [35]. PP2C is a negative regulatory element that normally binds to the ABA receptor protein, leaving the ABA receptor protein in an inhibited state [36]. CPK20 showed a decreasing expression trend in GUIGE8, while the opposite was true in GUIGE18 after SpM infection (Figure 9B). RVE8 showed an increasing expression trend in GUIGE8, while the opposite was true in GUIGE18 after SpM infection (Figure 9C). Expression of this gene was significantly lower at 3 dpi in the GUIGE8 than in GUIGE18. However, the expression level of the ABF-encoded genes (ABF4) had significantly increased at both 1 and 3 dpi after SpM infection in GUIGE8 (Figure 9F), indicating that the transcript level of the susceptible cultivar was activated by SpM infection. These results indicate that the up-regulation of genes encoding the ABA receptor was associated with the resistance of pueraria to the SpM pathogen. SA played a positive regulatory role in GUIGE8 during the SpM infection, while JA and ABA played a negative regulatory role.

Early studies have found that JA signal transduction has two resistance pathways. One is related to an Eth-mediated resistance pathway, and the other is negatively related to the SA pathway [37,38,39]. When plants are infected by pathogens, salicylic acid-mediated allergic reactions and systematically acquired resistance enable plants to obtain certain resistance [40,41]. It has been found that the activation of ABA signal occurs in the early stage of plant infection. Citrus canker infection enhances ABA biosynthesis by inducing NCED gene expression, thus promoting JA accumulation and inhibiting effective SA-mediated defense [42]. ABA plays a vital role in plant responses to biotic stresses [43]. ABA receptors will form a complex to mediate ABA signaling. ABA also induces an increase in intracellular calcium ion concentration [44]. Ca^2+^ usually acts as an intracellular secondary messenger that activates protective enzymes to alleviate the damage caused by plant diseases [45]. When the intracellular Ca^2+^ concentration increases, it promotes Ca^2+^ binding, thereby activating them, after which calcium-binding proteins indirectly generate ROS, which further activate the MAPK cascade reaction [46]. Many studies have found that ROS eruption in plant cells may be the core downstream event of Ca^2+^ influx induced by disease-resistant bodies [47,48,49].

Many experiments have found that secondary metabolites can be rapidly synthesized and inhibit the invasion of pathogens [50,51,52]. Flavonoids are the main secondary metabolites and phytotoxins of pueraria, which significantly changes after SpM infection to improve its defense ability. In Our study, the total flavone and isoflavone content in the two varieties showed no difference at 0 dpi, while its content increased significantly from 1 dpi to 3 dpi (Figure 10). There was also more accumulation in GUIGE8 compared to GUIGE18. The flavonoid biosynthetic process (GO: 0009813), isoflavonoid metabolic process (GO: 0046287), and isoflavonoid biosynthetic process (GO: 0009717) were found to be significantly enriched in GUIGE8. Five genes with expression related to the flavonoid biosynthetic process were analyzed (Figure 11). The results showed that UGT73C2, IFS2, and AS2 were up-regulated, while CHS1 and CHI were down-regulated in the two cultivars. In addition, the key synthetase CHI, CHS1, and IFS2, which are involved in the flavonoid biosynthetic process, were expressed at a higher level in GUIGE8 at 1 and 3 dpi compared to GUIGE18. This indicates that the reaction of flavonoid biosynthesis is more intense in susceptible cultivar after SpM infection. It is speculated that the susceptible cultivar may increase resistance to SpM through the accumulation of flavonoids.

**Figure 12:**
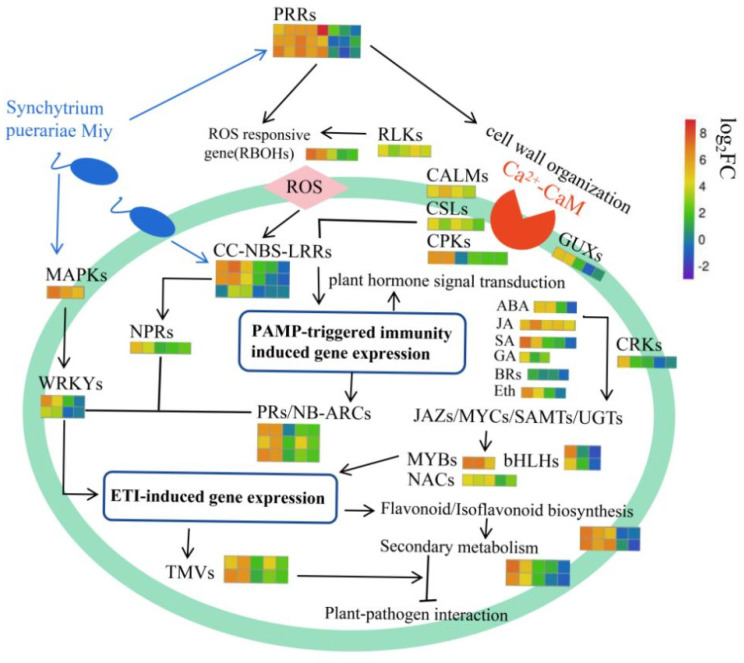
Hypothetical model of resistance to SpM (*Synchytrium puerariae Miy)* mechanism related DEGs in *Pueraria Lobata* based on transcriptomics analyses.

It has become a hot research topic to use high-throughput sequencing technology to analyze transcriptome data of plants and to mine metabolic pathways and regulatory mechanisms [53,54,55]. Based on cytological evidence of the interaction between legume plants and pathogens and drawing on immunological research on model plants, a model diagram of the regulation mechanism of activation of pueraria resistance after SpM infection was proposed with transcriptome data analysis (Figure 12).

This comparative transcriptome analysis of pueraria [*Pueraria lobata (Willd.) Ohwi*] preliminarily reveals candidate genes and pathways involved in pseudo-rust resistance. Utilizing effective defense pathways comprising a complex resistance network is necessary for pueraria in response to SpM infection. Moreover, further investigations will be focused on functional validation of the selected DEGs. The mechanism of resistance is still unclear and requires further experiments and exploration.

## 4. Materials and Methods

### 4.1. Plant Materials and Pathogen Infection

Pueraria cultivars, GUIGE18 (pseudo-rust disease resistant) and GUIGE8 (pseudo-rust disease susceptible) were used for inoculation with SpM in this study. After molecular identification of SpM collected from Guangxi South Subtropical Agricultural Science Research Institute (Supplementary Figure S1), a suspension of 1 × 10^6^ sporangia/mL was prepared with sterile water for reserve. Two pueraria cultivars were grown in a greenhouse under a controlled temperature of 24–28 °C, with an average humidity of 60%. After 30 days of growing, at the 5–8 leaves stage, and when plants were approximately 25–30 cm high, leaves from three plants of each cultivar were inoculated with SpM by suspension spraying. Three plants of each cultivar were mock inoculated with sterile water to use as negative controls. To clarify the changes in the gene expression levels of GUIGE18 and GUIGE8 during the first stages of their compatible interactions with SpM, leaf samples were taken before infection (0 days, control) and then again after 1 and 3 days, respectively. Three biological replicates were prepared at each time point. In total, 18 samples were immediately frozen in liquid nitrogen and stored at −80 °C for total RNA isolation and further analysis.

### 4.2. RNA Extraction, Library Construction, and RNA Sequencing

Total RNA was extracted using Trizol reagent (Invitrogen, CA, USA) following the manufacturer’s procedure. The total RNA quantity and purity were analyzed with a Bioanalyzer 2100 and an RNA 1000 Nano LabChip Kit (Agilent, CA, USA), with RIN number >7.0. Poly(A). RNA was purified from total RNA (5 ug) using poly-T oligo-attached magnetic beads using two rounds of purification. Following purification, the mRNA was fragmented into small pieces using divalent cations under an elevated temperature. Then the cleaved RNA fragments were reverse-transcribed to create the final cDNA library in accordance with the protocol for the mRNA-Seq sample preparation kit (Illumina, San Diego, CA, USA). We then performed the paired-end sequencing on an Illumina Novaseq™ 6000 (LC Sciences, Houston, TX, USA), following the vendor’s recommended protocol. The sequencing work was commissioned by Lianchuan Biotechnology Co., LTD, Hangzhou, China.

### 4.3. De Novo Assembly, Unigene Annotation, and Functional Classification

Firstly, Cutadapt [56] and in-house perl scripts were used to remove the reads that contained adaptor contamination, low quality bases, and undetermined bases. Sequence quality was then verified using FastQC (http://www.bioinformatics.babraham.ac.uk/projects/fastqc/ accessed on 19 March 2021), including the Q20, Q30, and GC-content of the clean data. All downstream analyses were based on clean data of high quality. De novo assembly of the transcriptome was performed with Trinity 2.4.0 [57]. All assembled unigenes were aligned against the non-redundant (NR) protein database (http://www.ncbi.nlm.nih.gov/, accessed on 25 March 2021) and eggNOG (http://eggnogdb.embl.de/, accessed on 25 March 2021) databases using DIAMOND [58], with a threshold of E value < 0.00001.

### 4.4. Sequencing Analysis and Differential Expression Analysis

Based on the same dataset, a clustering analysis was also performed using hclust from the stats package in R (http://www.R-project.org/, accessed on 22 March 2021) to detect clusters among all the 18 samples. Read counts per gene were expressed as the expected number of fragments per kilobase of transcripts per million mapped fragments (FPKM), and unigene abundance differences between the samples were calculated based on the ratio of the FPKM values and false discovery rate (FDR). Genes with |log2(FoldChange)| > 2, FDR ≤ 0.05, and FPKM ≥ 10 were considered DEGs [59]. GO classification was performed via Gene ontology, (http://www.geneontology.org/, accessed on 24 March 2021), and the GO distributions of DEGs were then obtained from three levels: biological process, cellular component, and molecular function. KEGG analysis was performed via the Kyoto Encyclopedia of Genes and Genomes pathway, (http://www.genome.jp/kegg/, accessed on 26 March 2021). A corrected *p*-adjust ≤ 0.05 was chosen as the threshold value for determining significantly enriched GO terms and KEGG enrichment pathways.

### 4.5. Measurements of Hormones and Defense Enzyme Activity

Endogenous hormone contents, including abscisic acid (ABA), jasmonic acid (JA), and salicylic acid (SA), were determined using an ultra-performance liquid chromatography-tandem mass spectrometry (UPLC-MS) system for samples of GUIGE18 and GUIGE8. PPO, CAT, and SOD activities were measured using kits (Shenggong Biological Co., LTD, Shanghai, China), and specific operations were carried out according to the kit instructions.

### 4.6. Quantitative Real-Time PCR (qRT-PCR) Analysis

Total RNA isolation, DNase I digestion, and first-strand cDNA synthesis were performed as previously described. The qRT-PCR was carried out on the Biometra TOne 96 PCR System (Jena, Germany) with the PrimeScript^TM^ RT reagent Kit with gDNA Eraser (perfect real time, RR047) and TB Green Premix Ex Taq^TM^ Ⅱ (Tli RnaseH Plus, RR820) (Takala, Beijing, China). Primer pairs were designed to amplify the actin gene, along with the nine genes involved in different pathways, using Primer Premier (v5.0) (Supplementary Table S2). All reactions were prepared in a final volume of 10 µL (containing 1 µL of cDNA, 1 µL of each specific primer, 5 µL of TB Green Premix Ex Taq^TM^ Ⅱ, and 2 µL of RNase-free water). β-actin was used as the internal control. The mRNA relative expression of each gene was calculated using the 2^−^^△△Ct^ method. Every target gene was run and analyzed in three biological replicates by one-way analysis of variance. *p*-values < 0.05, calculated using Dunnett’s test, was regarded as statistically significant.

### 4.7. Statistical Analysis

The statistical analysis was conducted with SPSS.22 for Windows. One-Way ANOVA analysis and Tukey’s honestly significant difference test were performed to test for differences between the means of the data for GUIGE18 and GUIGE8 at different stages using a significance level of *p*-values < 0.05. Figures were made with GraphPad Prism 6 (San Diego, CA, USA).

## 5. Conclusions

In this study, a total of 3617 and 8554 DEGs were identified in the susceptible pueraria cultivar (GUIGE8) and the resistant pueraria cultivar (GUIGE18), respectively, in three treatment groups after SpM infection. A total of 7044 DEGs were identified for their potential role between the two cultivars, of which 406 co-expressed genes were identified in the three comparison groups. Further analysis showed that 8, 8, and 5 of the identified DEGs were primarily involved in the oxidation-reduction process, plant hormone signal transduction, and the flavonoid biosynthetic process, which related to pueraria’s resistance to pseudo-rust. Furthermore, the GO and KEGG enrichment analysis suggested that MAPK signaling pathway-plant, cell wall organization, starch and source metabolism, protein phosphorylation, a secondary metabolite biosynthesis process, and a defense response may play vital roles in SpM resistance. The activities of defensive enzymes (CAT, SOD, and PPO) were increased after SpM infection, and its interaction may alter energy metabolism to maintain ROS balance. Higher contents of ABA, SA, and JA in GUIGE18 (resistant cultivar) after SpM infection might mainly contribute to the resistance. Meanwhile, the rapid accumulation of total flavones and isoflavones of GUIGE8 (susceptible cultivar) may explain its improved performance under pueraria pseudo-rust stress.

## Figures and Tables

**Table 1:** Database Annotation Result Statistics.

Database	Number	Ratio%
GO	32,304	45.57
KEGG	25,504	35.97
Pfam	30,022	42.35
swissprot	26,129	36.86
eggNOG	39,537	55.77
KOG	25,504	35.97
NR	39,828	56.18
Intersection	7445	10.50
Overall	59,701	84.21

## Data Availability

Data available on request due to restrictions eg privacy or ethical. The data presented in this study are available on request from the corresponding author. The data are not publicly available due to the research result have not yet been published.

## Supplementary Materials

The following supporting information can be downloaded at https://www.mdpi.com/article/10.3390/ijms23095223/s1.
