# Supplementary material for: Comparative Transcriptome Analysis Provides Insights into the Resistance in Pueraria [Pueraria lobata (Willd.) Ohwi] in Response to Pseudo-Rust Disease
Source: Int J Mol Sci. 2022 May 7;23(9):5223. doi: 10.3390/ijms23095223 (PMC9101505; doi:10.3390/ijms23095223)
Supplement: Supplementary file 1 [file ijms-23-05223-s001.zip › ijms-1706530-supplementary.pdf]

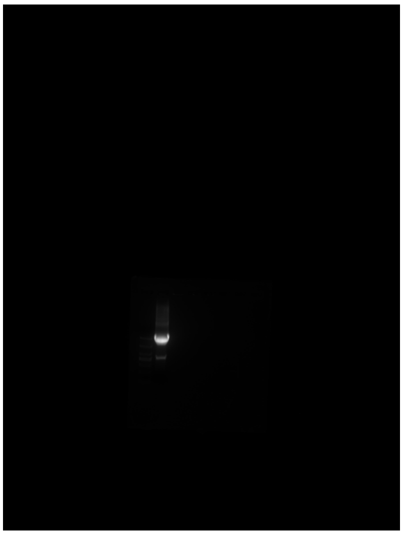

(**A**)

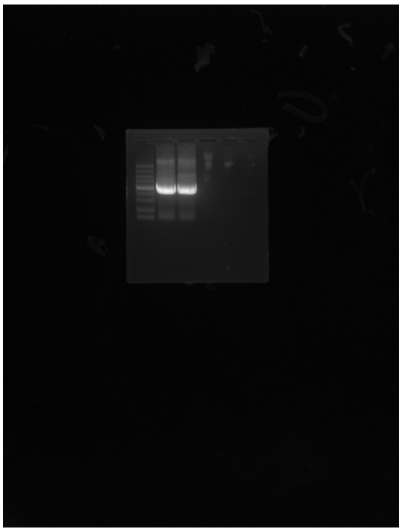

(**B**)

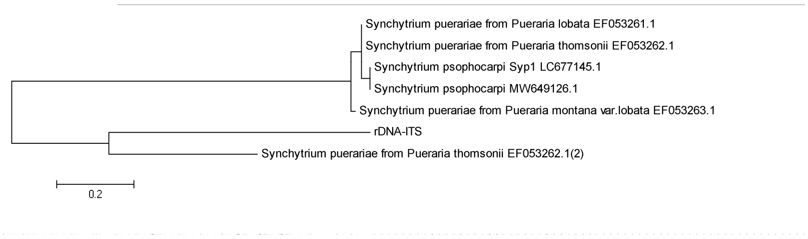

(**C**)

Figure S1. (**A**): Gel analysis on pathogen DNA extraction; 1: DNA extraction product of SpM pathogen; (**B**): Gel analysis on PCR amplification; 1: PCR amplification product of SpM pathogen DNA; M: DL5000 marker; (**C**):Verification results of SpM pathogen.

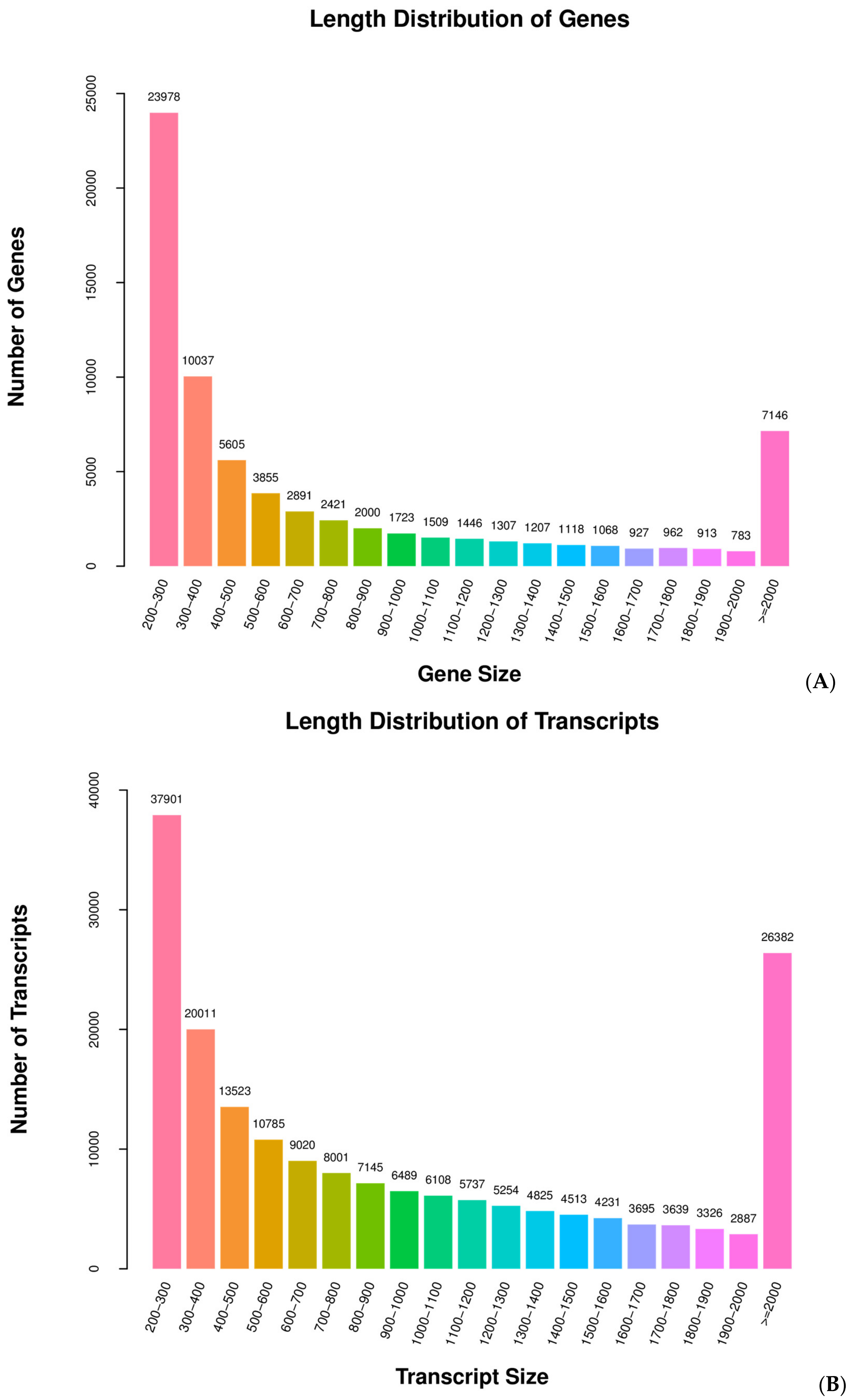

Figure S2. (**A**) Length Distribution of Gens; (**B**) Length Distribution of Transcripts.

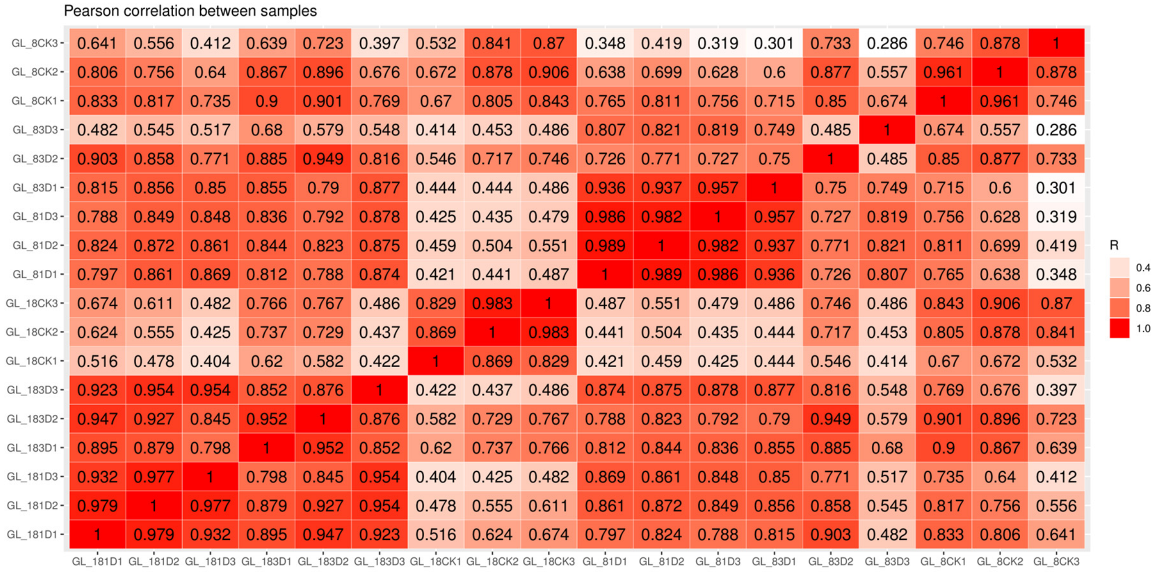

Figrue S3. Pearson Correlation between Samples.

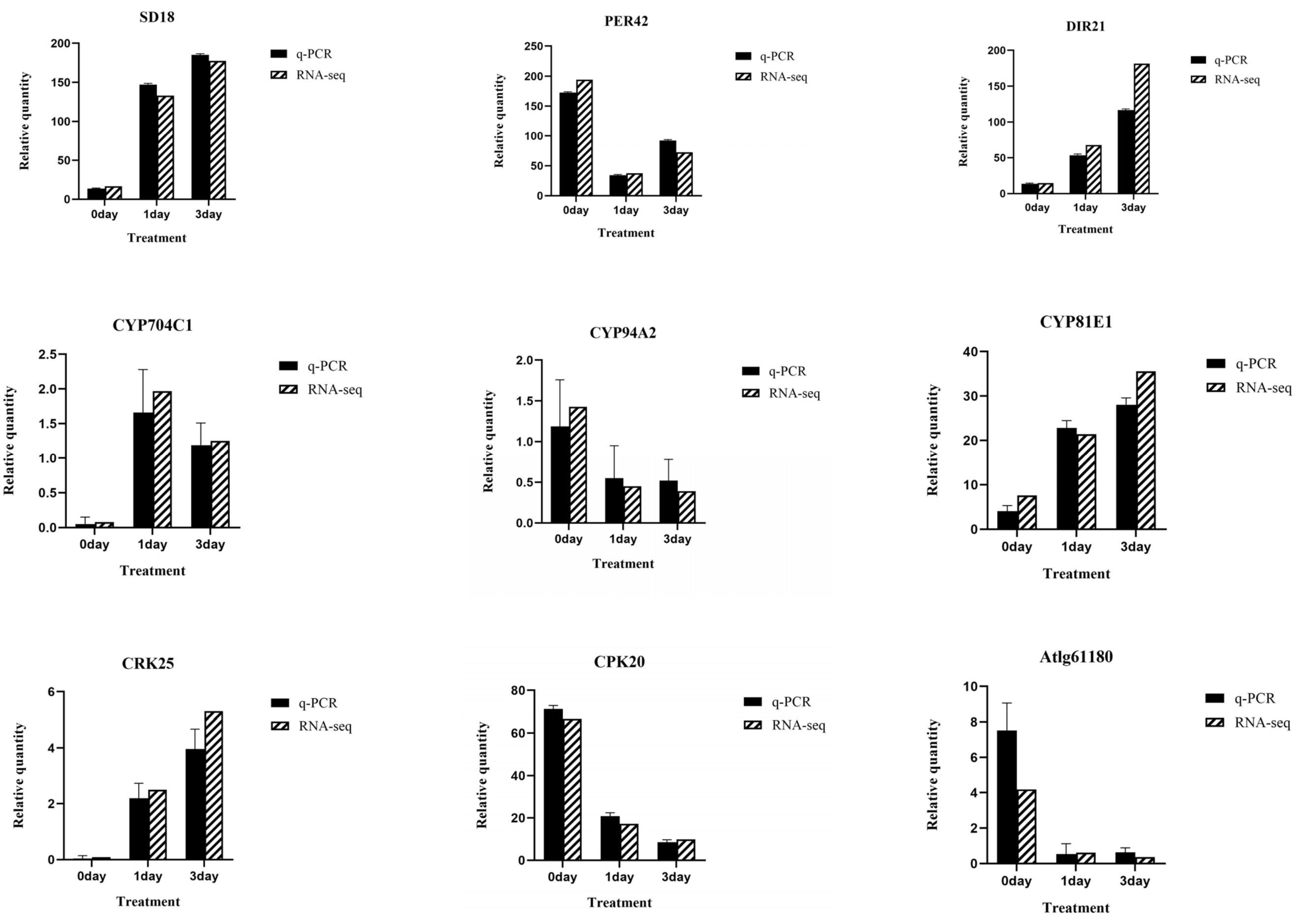

Figure S4. The analysis of the expression levels of the nine unigenes between the q-PCR and RNA-seq.

Supplementary Table S1.Quality statistics of transcriptome sequenceing after filtering.

Sample	Raw reads	Clean reads	Clean reads ratio/%	Q20 ratio/%	Q30 ratio%	GC ratio%
GL_181D1	50,857,956	48,560,756	95.48	98.49	94.97	46.11
GL_181D2	46,251,570	43,374,130	93.78	98.59	95.20	45.91
GL_181D3	47,639,518	45,307,178	95.10	98.56	95.12	45.56
GL_183D1	36,838,316	33,488,514	90.91	98.59	95.22	46.03
GL_183D2	47,537,152	43,101,554	90.67	98.61	95.27	46.87
GL_183D3	37,285,532	35,638,936	95.58	98.67	95.43	45.98
GL_18CK1	39,286,022	31,531,224	80.26	98.60	95.27	45.58
GL_18CK2	40,095,814	34,673,632	86.48	98.55	95.13	45.68
GL_18CK3	38,000,458	28,712,176	75.56	98.54	95.12	45.53
GL_81D1	47,179,536	44,539,400	94.40	98.58	95.18	45.10
GL_81D2	46477516	43,986,328	94.64	98.51	95.00	45.08
GL_81D3	34,237,314	32,805,196	95.82	98.57	95.15	45.03
GL_83D1	45,219,580	42,858,066	94.78	98.55	95.14	45.44
GL_83D2	34,559,698	32,980,312	95.43	98.61	95.25	46.13
GL_83D3	45,889,650	42,035,796	91.60	98.47	94.93	44.69
GL_8CK1	43,006,176	40,583,826	94.37	98.47	94.90	45.55
GL_8CK2	38,947,166	33,551,570	86.15	98.57	95.23	45.98
GL_8CK3	56,103,522	55,649,222	99.19	98.46	94.98	46.27

Supplementary Table S2.Primers for real-time quantative PCR.

Gene	Foward primer(5'→3')	Reverse primer(5'→3')
Actin	TCACCAGAATCAAGCACAATACC	TGCCCCTGAAGAACACCCT
CYP81E1	CTCAACCCAACGCATCCA	CCCCGACAGCATCCTCAT
CYP704C1	GGAAAATCAATAGGGGACC	AGCCAGTAGCCGAAAAGT
DIR21	TTGGTGGAACAGGTGCTT	GCCCTCGTGAGATGAATA
CRK25	GTAACAAAACCTTCGCAGTC	GGTGGCGTTGGAAGATAG
SD18	GATGACATTGATTTACCGAC	ACACTTCTCCAAAGCCAC
CYP94A2	CTCCCAAAGCATACCCTA	TTGAGAATGTGCTGGACC
PER42	CTGAAGAAGTGCCCTGAT	TGTCCAATATGTTCCTGTAGTA
CPK20	TCCTCTTTCGTTTCCGTC	ACCCTCCTCACCACTCCT
At1g61180	AAGAACACGCTTGTAATCC	CTCACCCCTTTTGTCGTA
